# A supervised deep neural network approach with standardized targets for enhanced accuracy of IVIM parameter estimation from multi‐SNR images

**DOI:** 10.1002/nbm.4774

**Published:** 2022-06-06

**Authors:** Alfonso Mastropietro, Daniel Procissi, Elisa Scalco, Giovanna Rizzo, Nicola Bertolino

**Affiliations:** ^1^ Istituto di Tecnologie Biomediche Consiglio Nazionale delle Ricerche Segrate Italy; ^2^ Department of Radiology Northwestern University Chicago Illinois USA

**Keywords:** deep learning, deep neural network, diffusion‐weighted magnetic resonance imaging, intravoxel incoherent motion, IVIM

## Abstract

Extraction of intravoxel incoherent motion (IVIM) parameters from noisy diffusion‐weighted (DW) images using a biexponential fitting model is computationally challenging, and the reliability of the estimated perfusion‐related quantities represents a limitation of this technique. Artificial intelligence can overcome the current limitations and be a suitable solution to advance use of this technique in both preclinical and clinical settings. The purpose of this work was to develop a deep neural network (DNN) approach, trained on numerical simulated phantoms with different signal to noise ratios (SNRs), to improve IVIM parameter estimation. The proposed approach is based on a supervised fully connected DNN having 3 hidden layers, 18 inputs and 3 targets with standardized values. 14 × 10^3^ simulated DW images, based on a Shepp–Logan phantom, were randomly generated with varying SNRs (ranging from 10 to 100). 7 × 10^3^ images (1000 for each SNR) were used for training. Performance accuracy was assessed in simulated images and the proposed approach was compared with the state‐of‐the‐art Bayesian approach and other DNN algorithms. The DNN approach was also evaluated in vivo on a high‐field MRI preclinical scanner. Our DNN approach showed an overall improvement in accuracy when compared with the Bayesian approach and other DNN methods in most of the simulated conditions. The in vivo results demonstrated the feasibility of the proposed approach in real settings and generated quantitative results comparable to those obtained using the Bayesian and unsupervised approaches, especially for *D* and *f*, and with lower variability in homogeneous regions. The DNN architecture proposed in this work outlines two innovative features as compared with other studies: (1) the use of standardized targets to improve the estimation of parameters, and (2) the implementation of a single DNN to enhance the IVIM fitting at different SNRs, providing a valuable alternative tool to compute IVIM parameters in conditions of high background noise.

## Graphical abstract

We developed a supervised DNN approach to improve IVIM parameter estimation. The method outlines two innovative features: (1) the use of standardized targets and (2) the implementation of a single DNN to enhance the IVIM fitting at different SNRs, providing a valuable alternative tool to compute IVIM parameters.

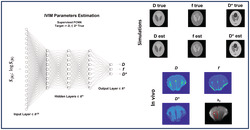

**Abbreviations**
AI: artificial intelligence
ANN: artificial neural network
DNN: deep neural network
DW: diffusion weighted
FR: failure rate
IVIM: intravoxel incoherent motion
MAD: median absolute deviation
MdAE: median absolute error
MdB: median bias
RCV: robust analog of the coefficient of variation
ROI: region of interest
SNR: signal to noise ratio

## 1 INTRODUCTION

Intravoxel incoherent motion (IVIM) is an MRI technique in which a bi‐exponential representation of diffusion‐weighted (DW) images acquired with several different *b*‐values can account for both diffusion and perfusion tissue properties.^1^, ^2^, ^3^ Use of the IVIM technique has significant potential in both clinical and preclinical applications focusing on oncological,^4^, ^5^ neurovascular disease^6^, ^7^, ^8^ and other physio‐pathological conditions.^9^, ^10^, ^11^ The main features which determine the diagnostic and research potential of the IVIM method are (1) the widespread availability of DWI sequences on both preclinical and clinical scanners and (2) the non‐invasive nature of the technique, which does not require administration of the exogenous contrast agents normally used for the determination of tissue perfusion.

However, the ability of IVIM to generate reliable quantitative parametric images reflecting actual tissue perfusion is dependent on the quality of the computation, provided by the fitting algorithms used to extract them from the acquired MRI data. The accuracy in estimation of IVIM parameters is particularly sensitive to the fitting approach, which often yields sub‐optimal results, especially for perfusion‐related parameters when dealing with high‐background‐noise MR images. In many such cases the quality of the estimated maps is poor^12^, ^13^, ^14^ strongly impacting their practical utilization. There is extensive literature exploring the reliability of the perfusion values generated with the IVIM method and comparing the different possible approaches^15^, ^16^, ^17^ to try to improve them. Among these, Bayesian approaches based on probabilistic algorithms seem to provide better performance overall when compared with the standard least‐square methods in terms of both reproducibility and accuracy. However, the probabilistic approach is also more computationally demanding, requiring longer computation times,^18^, ^19^ which also limits their practical use.

It is widely recognized that medical image processing and analysis supported by artificial intelligence (AI) offers a range of new powerful and versatile tools that are suitable for advancing medical imaging analysis pipelines.^20^ It is therefore valuable to explore the potential of AI methods to overcome the limitations of the algorithms currently employed for IVIM parameter estimation. Among AI algorithms, deep neural networks (DNNs) are widely popular because of their flexibility, which makes them suitable for solving non‐trivial fitting problems such as the ones encountered when attempting to generate physiologically reliable quantitative maps from IVIM data. One of the major advantages of DNNs is the fact that they can be deployed for data analysis immediately after the training process and provide extremely fast and efficient processing timelines. In this perspective, DNNs are a tool worth investigating to advance the reliability of quantitative IVIM maps and boost their use in the medical field.

Two possible strategies offering both advantages and disadvantages to overcome this issue are generally described in the existing literature.^21^, ^22^, ^23^ These strategies focus on implementation of (i) a method that uses supervised machine learning utilizing a loss function linked to the quantitative error in parameter estimation and (ii) a method based on an unsupervised approach with a loss function based on the quantitative error in the original input signal.^24^

The supervised approach was first developed by Bertleff et al.^21^ In their work, they used an artificial neural network (ANN) trained in a simulated dataset for the estimation of a joint IVIM–kurtosis model obtaining better performance than least‐square fitting. However, the approach was not properly optimized, and this could lead to biased estimates of IVIM parameters. Furthermore, different ANNs were used when dealing with images presenting different levels of signal to noise ratio (SNR) (i.e., a requirement to determine an optimal specific ANN for each level of SNR that only worked with the associated set of images), thus making it difficult to select an optimal and general solution that could be used practically.

More recently, the use of an unsupervised approach was first proposed by Barbieri et al.^22^ and further improved by Kaandorp et al.^23^ They trained and tested unsupervised DNNs with simulated data and in vivo images, finding improvements compared with standard approaches. The unsupervised approaches are capable of training directly on real data with no ground truth and a priori knowledge; however, this means that, to exploit their whole potential, they are strongly dependent on the available number of data and require to be retrained if new data are added (e.g., from different scanners) or if other anatomical regions are imaged even when the scanning protocols remain the same.

To improve the performances of the current AI methods and overcome some of their limitations, starting from the study by Bertleff et al., this work aims to develop a fully connected DNN trained on numerical phantoms for IVIM map computation. The main innovation of the proposed approach is based on the use of standardized targets, which improves the estimation of the parameters, along with the use of a single DNN for fitting signals at different SNRs.

Performance testing of the proposed supervised approach was achieved through comparison with a state‐of‐the‐art Bayesian method^25^ and with other AI algorithms proposed in the literature, such as the supervised approach proposed by Bertleff et al.^21^ and the unsupervised method proposed by Kaandorp et al.^23^ We also tested the performance of the trained DNN using data acquired from live animals (mice) in a high‐field scanner (7 T) to evaluate the suitability of our method in realistic scenarios.

## 2 METHODS

### 2.1 Simulations

To train, validate and test the performance of the proposed supervised DNN algorithm in a controlled ideal scenario we applied our method on numerical phantoms. Numerical phantoms were generated using a MATLAB (R2020a—MathWorks, Natick, MA, USA) custom‐made script. Considering wide ranges of diffusion (*D*) [0.0005–0.002 mm^2^/s], perfusion fraction (*f*) [0.025–0.4] and pseudo‐diffusion (D*) [0.005–0.1 mm^2^/s] values, which are in agreement with previous works,^21^, ^22^ DW images were generated at different *b*‐values (0, 25, 50, 75, 100, 150, 300, 800, 1000 s/mm^2^) using the formula

$$S_{b}=S_{0}\left(1-f\right)e^{-\mathrm{bD}}+S_{0}f\;e^{-bD^{*}}.$$

The signal of the simulated images was corrupted by Rician noise to create the DW images at different SNRs (10, 15, 20, 25, 50, 75, 100). This was performed by adding the noise contribution given by the addition of two Gaussian noises (GNs) as follows:

$$S\left(i,j\right)=\sqrt{{\left[S_{\infty }\left(i,j\right)+\mathrm{GN}\right]}^{2}+{\mathrm{GN}}^{2}}$$

where *S*_∞_ denotes the simulated noiseless value and each GN contribution is independently sampled from a Gaussian distribution with a null mean value and variance equal to 1/SNR.^2^

To increase the complexity of the simulated dataset, a Shepp–Logan phantom (size 64 × 64 pixels) having six different regions of interest (ROIs) along with background noise was used as a template for our simulations; each ROI was characterized by a randomly generated (from a uniform distribution) parameter triplet and different *b*_0_ values. SNR was defined voxel‐wise and scaled considering changes in *S*_0_ to guarantee the same SNR over a single simulated phantom.

**FIGURE 1:**
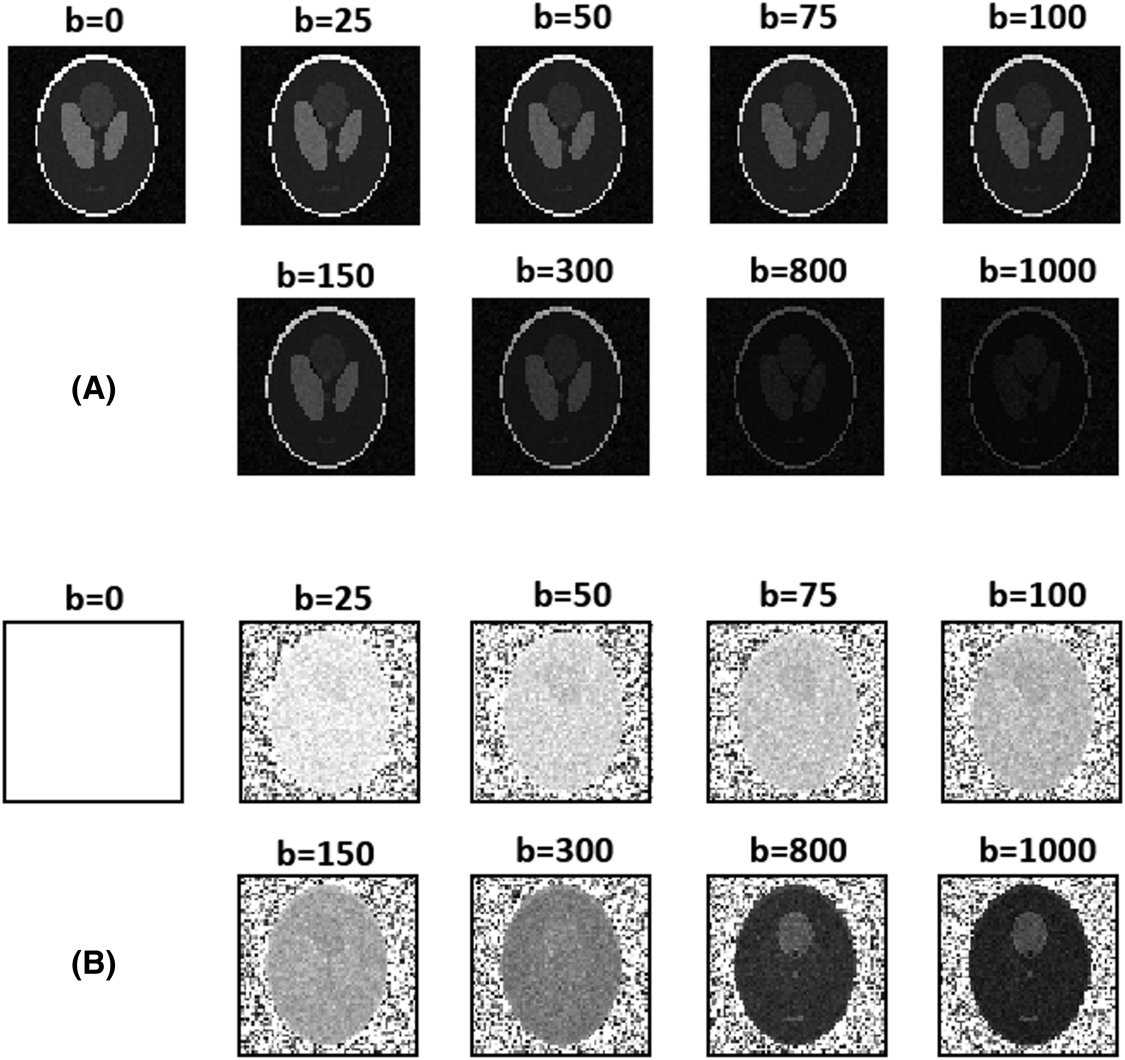
A, An example of simulated DW images obtained at SNR 25. B, The same DW images normalized with respect to *b*_0_

A total of 2000 phantoms were generated for each SNR. Some examples of simulated datasets are shown in Figure 1.

### 2.2 Tuning of the supervised DNN approach

The proposed approach is based on a supervised fully connected network having as outputs the three IVIM parameters *D*, *f* and D*.

To optimize the final DNN architecture to be used we implemented training and testing of multiple DNN configurations using the MATLAB Machine Learning toolbox and simulated phantoms. The tested DNN configurations differed in the standardization of the targets, the number of the hidden layers and the number of input features as described below. A total of five DNN architectures were tested.

- To evaluate the effect of the number of hidden layers, three feedforward networks, with nine input nodes (one for each *b*‐value), having two, three or four hidden layers, were considered.
- To assess the effect of target standardization, two three‐hidden‐layer feedforward networks, with nine input nodes, with and without standardized targets were implemented. Since *D*, *f* and D* have ranges that differ significantly from each other (*D*, 10^−3^–10^−4^; *f*, 10^−2^; D*, 10^−2^–10^−3^), the standardization of the targets was performed to rescale and shift their values to have a mean of 0 and a standard deviation of 1 (unit variance). The output values, as estimated by the DNN, are then rescaled and reshifted into the original ranges to restore meaningful parameters.
- To test the effect of feature augmentation, a three‐hidden‐layer feedforward network, with standardized targets, having 18 input nodes was implemented. The nine additional input features were generated computing the natural logarithm of the original DW images.

The training was performed using a Levenberg–Marquardt backpropagation algorithm, and an adaptive learning rate was used with an initial value of 10^−3^, a decrease factor of 0.1 and an increase factor of 10. A hyperbolic tangent sigmoid function for the hidden layers and a linear activation function for the output layer were used as integral part of the network; the mean square error was chosen as a loss function. The network was trained using the first 1000 simulated images for each SNR (6000 images; more than 1 × 10^7^ signals), splitting the data randomly at 80%/20% for training/validation subsets. Normalized inputs (i.e., *S*_*b*_/*S*_*b*0_) were rescaled, mapping minimum and maximum values to [−1 1]. Background voxels were excluded from the training and the evaluation steps. The following stopping criteria were used: minimum gradient value 10^−7^; number of iterations 5000; validation checks 6.

### 2.3 In vivo animal data

For this experiment we imaged a female C7/BI6 mouse (age 4 months). The animal was anesthetized with a solution of oxygen and 2% isoflurane. During the scanning process animal respiration rate and temperature was monitored to assure its safety. Experimental procedures involving live animals complied with Northwestern's IACUC guidelines. MRI acquisitions were performed on a 7 T ClinScan MRI scanner (Bruker, Ettlingen, Germany) equipped with a 12 cm diameter gradient coil system (max strength 115 mT/m) using a four‐channel phased‐array receiver coil. A volume quadrature coil was used for transmission. The imaging protocol included multiple‐*b*‐value (*b* = 0, 25, 50, 75, 100, 150, 300, 800, 1000 s/mm^2^) SE‐EPI DW images (*T*_R_/*T*_E_ = 3500/27 ms, flip angle = 90°, averages = 4, slice thickness = 1 mm, voxel size = 0.282 × 0.282 mm^2^).

### 2.4 Performance analysis and metrics

The trained networks were used to compute *D*, *f* and D* of the remaining 1000 simulated images for each SNR. IVIM maps were also generated using a state‐of‐the‐art Bayesian method,^25^ an unsupervised neural network approach as proposed by Kaandorp et al.^23^ and a supervised method as proposed by Bertleff et al.^21^ For the implementation of the methods, the freely available codes provided by the authors were used for the Bayesian and the unsupervised approaches. Conversely, the supervised method was implemented as described in the original paper using a simplified model without the kurtosis parameter. The two DNN‐based approaches were trained for each specific SNR as described in the original papers. IVIM data were normalized to *S*_*b*0_ for both the Bayesian and unsupervised approaches, whereas they were just rescaled, mapping the minimum and maximum values to [−1 1], in the case of the supervised approach.

To assess the quality of the parameter estimates, the relative median absolute error (MdAE) and the relative median bias (MdB) were calculated as follows:

$$\text{MdAE}=\text{median}\frac{\left|P_{\mathrm{est}}-P_{\text{true}}\right|}{P_{\text{true}}}$$

$$\mathrm{MdB}=\text{median}\frac{P_{\mathrm{est}}-P_{\text{true}}}{P_{\text{true}}}$$

where **P**_true_ is the vector of ground truth parameters and **P**_est_ is the vector of the voxel‐wise estimated parameters over the whole simulations. MdAE and MdB were defined similarly to the metrics previously proposed by While,^16^ with the difference that the denominator, in our case, is represented by the ground truth values as proposed by Lanzarone et al.^18^

To quantify the precision and repeatability of the parameter estimates in homogeneous ROIs, a robust analog of the coefficient of variation (RCV)^26^ was used. RCV is defined as

$$\mathrm{RCV}=1.4826\frac{{\mathrm{MAD}}_{P\mathrm{est}}}{{\text{median}}_{P\mathrm{est}}}$$

where MAD is the median absolute deviation of the estimated parameters. RCV was calculated in each region separately and then the median value for all the simulations was considered.

Furthermore, the failure rate (FR), defined as the number of times the MdAE was greater than 50% within the overall number of signals, was considered as a further evaluation parameter.

The training time of the proposed approach was evaluated on a Dell Precision 7820 Tower (Intel Xeon Bronze 3204, 32 GB RAM, Ubuntu 20.04).

The five DNN performances were quantitatively compared using the proposed metrics to evaluate the best configuration. The selected configuration was finally compared with the other state‐of‐the‐art implemented methods.

The proposed method along with the other methods was also qualitatively and quantitatively evaluated on the in vivo mouse dataset to assess if the quality of the obtained results is in line with what we found in simulations.

### 2.5 Statistical approach

Considering that data do not have a Gaussian/normal distribution, we used the Kruskal–Wallis test, a non‐parametric version of one‐way ANOVA, to compare medians of the assessment metrics among the considered fitting approaches. Tukey's honestly significant difference procedure was used to correct for multiple comparisons. *p* < 0.05 was considered significant.

## 3 RESULTS

### 3.1 Tuning of the supervised DNN approach

**FIGURE 2:**
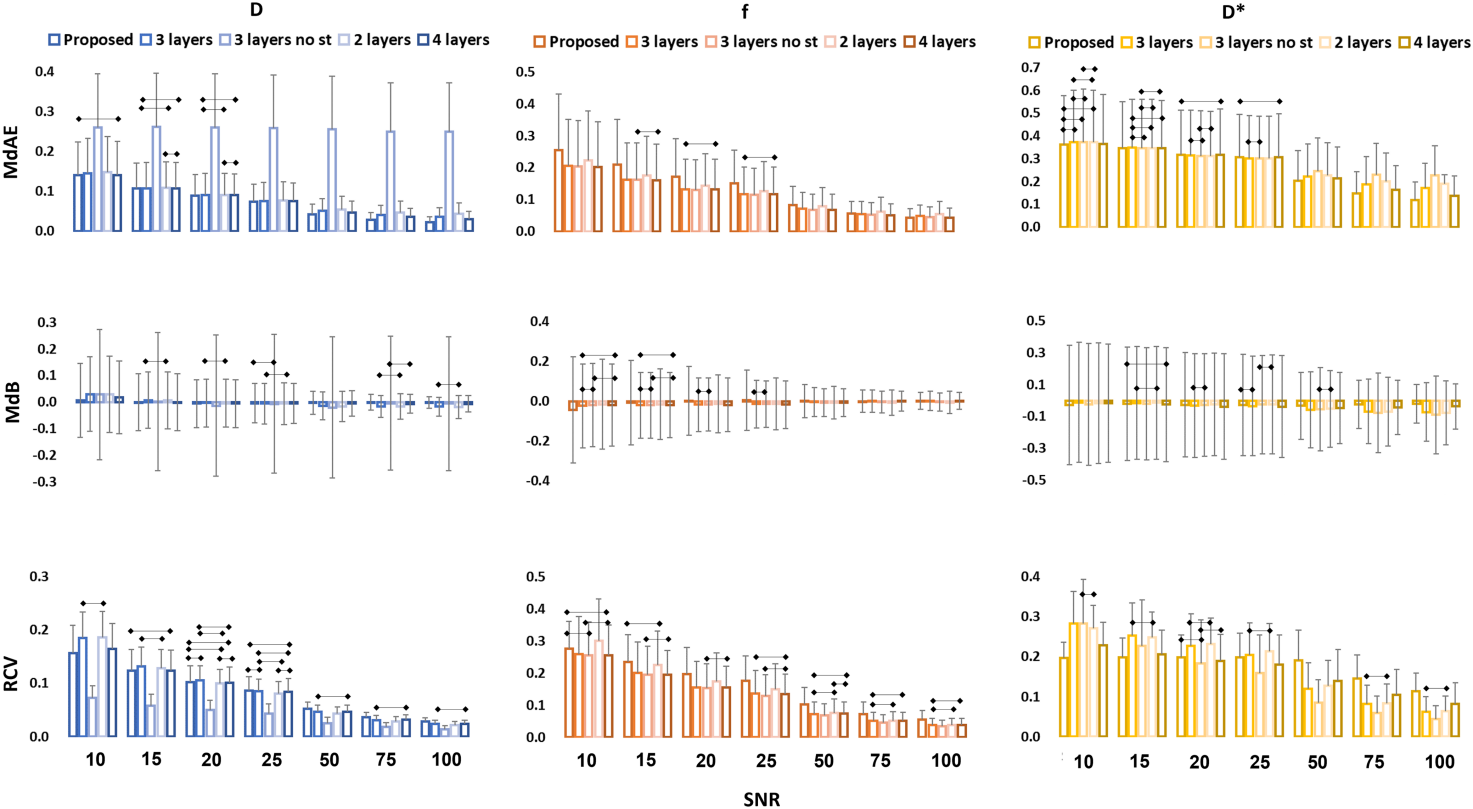
Quantitative comparison of the effects of different hidden layer numbers, use of standardization and log‐transformed inputs on the performance of DNN for the IVIM parameter estimation. Data are displayed as median ± MAD. Non‐significant differences are highlighted

**FIGURE 3:**
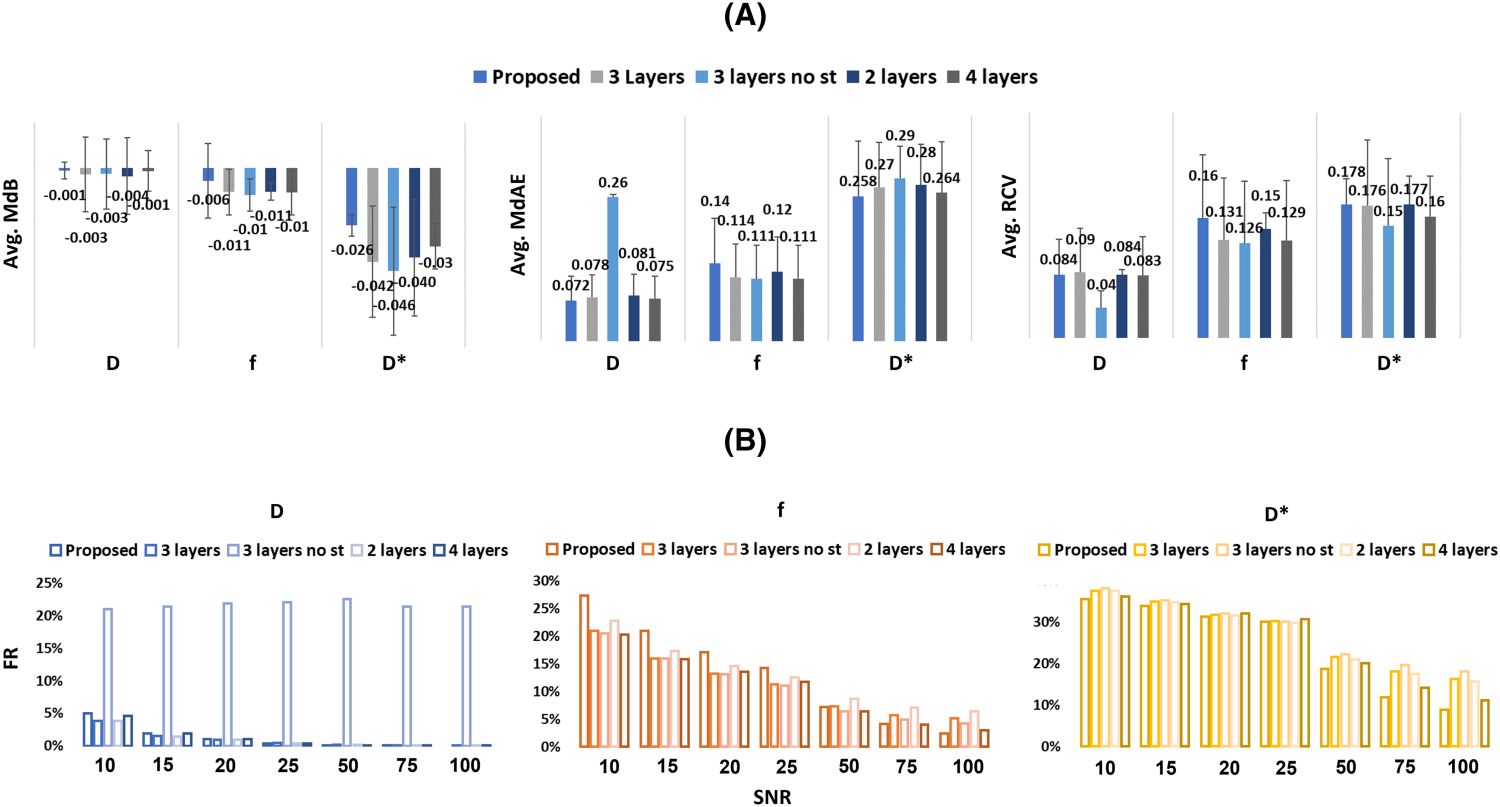
Quantitative comparison of the performances in the IVIM parameter estimation considering the average MdB, MdAE and RCV values (A) and the FRs (B) for different SNRs and different IVIM parameters

The analysis of the effect of input layers number on DNN performance shows overall minor effects (Figure 2 and Figure 3A). Looking at all different IVIM parameters—considering the assessment metrics MdAE, MdB, RCV—at different SNR levels, the four‐hidden‐layer DNN performs best in most of the conditions with average MdAEs of 0.075 ± 0.041 for *D*, 0.1106 ± 0.060 for *f* and 0.264 ± 0.091 for D*, average MdBs of −0.001 ± 0.009 for *D*, −0.011 ± 0.010 for *f* and −0.035 ± 0.010 for D* and average RCVs of 0.083 for *D*, 0.129 for *f* and 0.162 for D*; the three‐hidden‐layer DNN outperforms the two‐hidden‐layer DNN, especially for MdAE, on average and in most of the conditions, as displayed in Figures 2 and 3.

Considering the FR, the four‐hidden‐layer DNN is the best performing, with an average value of 12.4% whereas the three hidden layers slightly outperform the two hidden layers (13.2% versus 13.5%). More details on FR changes with SNRs for different parameters are displayed in Figure 3B.

The computational times for training two‐, three‐ and four‐hidden‐layer DNNs were about 2, 3.5 and 5.5 days respectively.

Target standardization decreases the MdAE, MdB, RCV and FR in *D* estimation significantly, increasing the measurement accuracy. Results show a large improvement in FR for *D*, going from above 20% to below 1% for SNR higher than 20. More subtle is the effect on *f* and D*, where the differences are mostly negligible. Accuracy appears slightly increased in the not standardized scenario for *f*, and in contrast slightly higher accuracy is shown with standardization for D* (Figures 2 and 3).

As shown in Figures 2 and 3, increasing the number of features from 9 to 18, including the natural logarithm of *S*_*b*0_ and DW signals *S*_*b*_, also leads to minor changes. Nevertheless, the 18‐feature configuration almost consistently improves, on average, the network accuracy (MdAE and MdB) when compared with the standardized three‐hidden‐layer DNN except for the MdAE for *f*.

Considering MdAE and MdB, in the simulated dataset, the three‐hidden‐layer DNN with 18 standardized inputs resulted overall in the best configuration, with a reasonable trade‐off between higher accuracy and lower computational training time. For RCV the proposed solution slightly underperforms with respect to the three‐hidden‐layer DNN, whereas regarding the FR, on average, the proposed solution shows slight improvement (12.9% versus 13.2%).

### 3.2 Proposed DNN architecture

**FIGURE 4:**
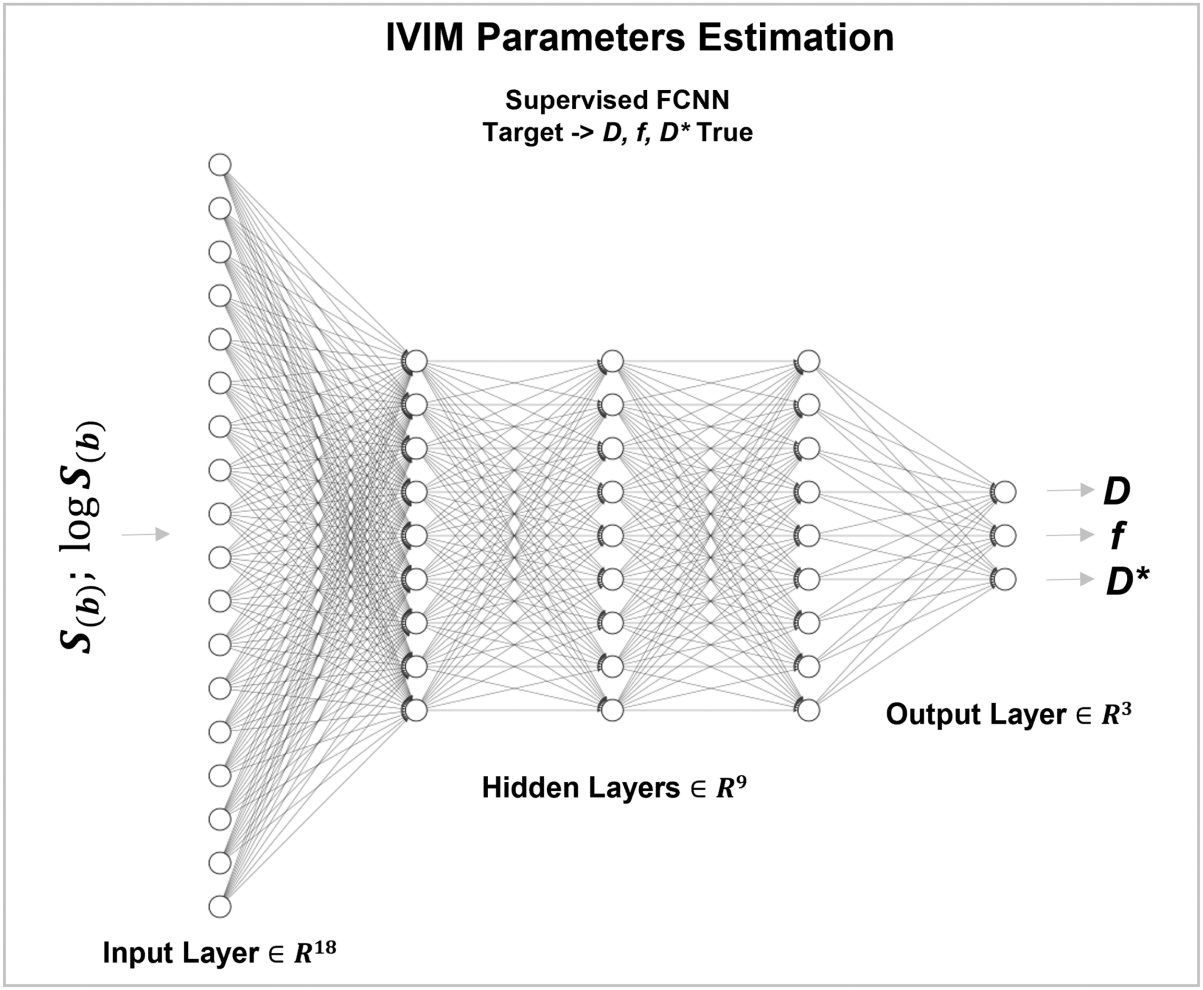
A sketch of the proposed DNN architecture having three hidden layers, two *b*‐value inputs and three standardized targets

Based on the results described above, the final proposed approach consists of a supervised fully connected DNN having 3 hidden layers, 18 inputs and 3 targets with standardized values. Shown in Figure 4 is a representation of the DNN architecture main frame used in this study.

### 3.3 Simulated dataset

**FIGURE 5:**
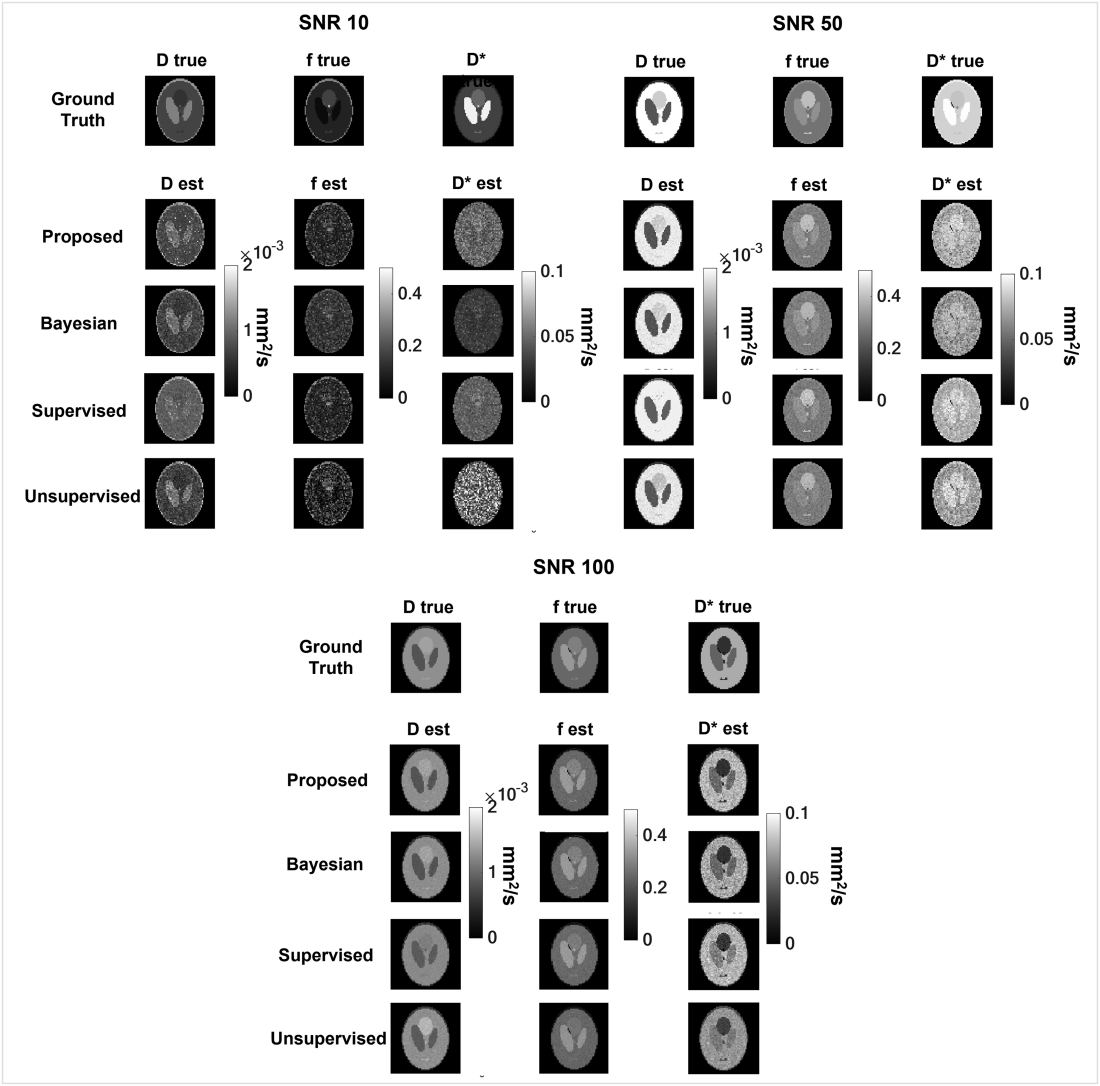
Some examples of estimated parameter maps obtained at different SNRs (10, 50, 100) using the proposed DNN approach and the other implemented methods

The performance of the optimized trained DNN was quantitatively evaluated in simulated datasets. In Figure 5 some examples of *D*, *f* and D* maps generated with the proposed method, as well as the other methods used for comparison, and their respective ground‐truth images are shown. Starting from a qualitative evaluation, as expected, SNR affects the performance of all the methods, with higher SNRs corresponding to better parameter estimation. *D* maps are, generally, those showing the best quality at each SNR, whereas D* maps are those that show the worst match with the ground truth and the highest variability among methods.

**FIGURE 6:**
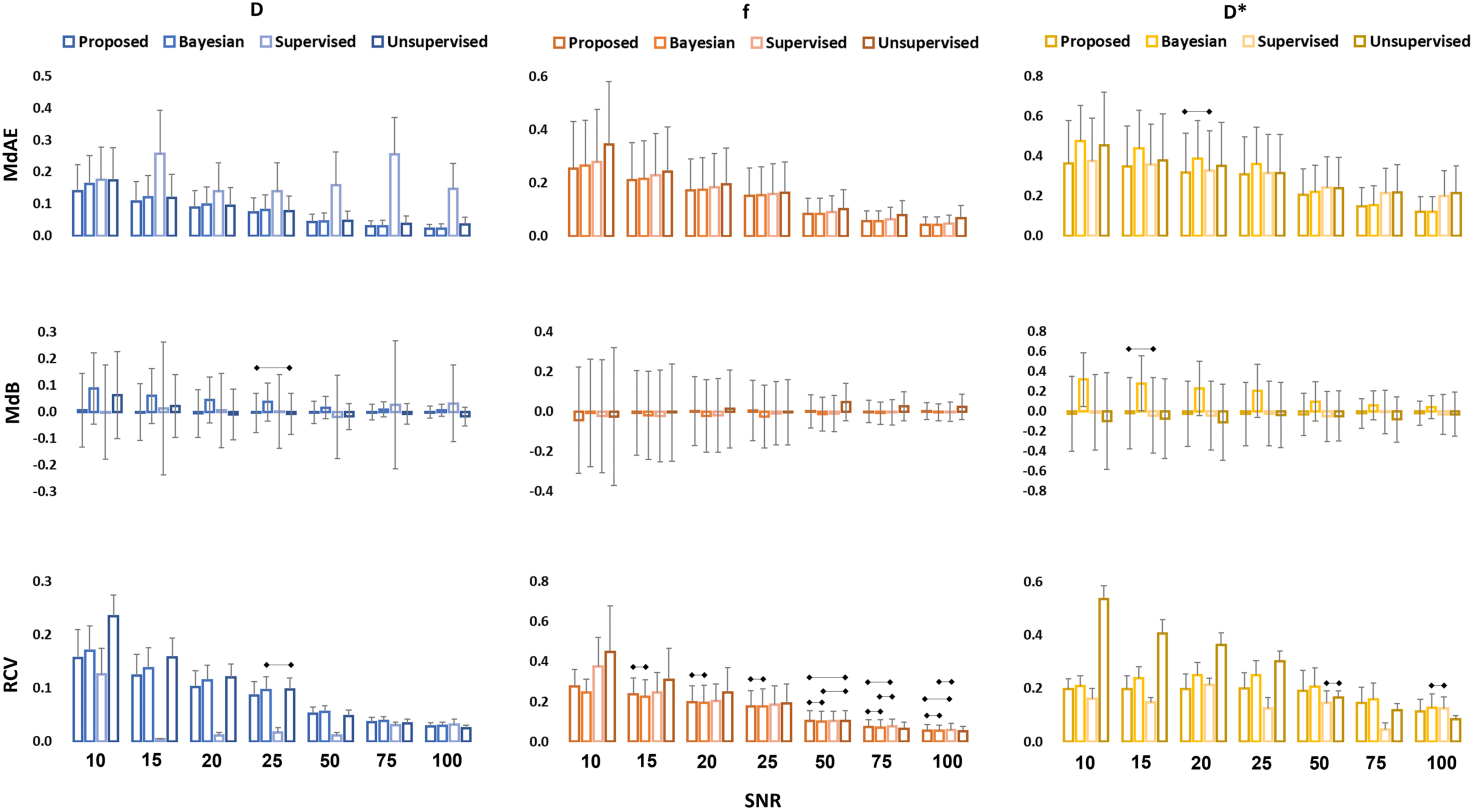
Metrics for performance assessment of the accuracy and precision of the implemented approaches in the estimation of IVIM parameters. Data are displayed as median ± MAD. Non‐significant differences are highlighted

In Figure 6 a quantitative comparison between our DNN approach and the other implemented methods is shown. Considering the MdAE, the proposed method is always the best performing in the case of *D* and D* estimation (14 out of 14), with values lower than 0.14 and 0.36 respectively. In the case of *f* estimation, our methods perform better than the other methods in five out of seven conditions (SNR 10, 15, 20, 25, 75), with a value lower than 0.25.

As for the MdB, the proposed method outperforms the others in four out of seven conditions (SNR 15, 50, 75, 100) in the case of *D*, with a median value lower than 0.006, and in four out of seven conditions (SNR 20, 50, 75, 100) in the case of *f*, with the highest value of 0.043 at SNR 10. In the case of D*, the proposed approach outperforms all the others in four out of seven conditions (SNR 15, 20, 50, 100).

Regarding the RCV, the proposed method has the second‐best performance for *D* and D* estimation, where it is slightly surpassed by the supervised approach, and it is still the second‐best performing method for *f*, where it is surpassed by the Bayesian method.

**FIGURE 7:**
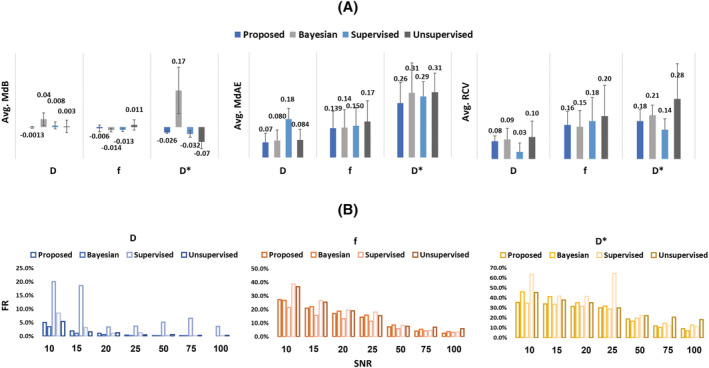
A, Summary of quantitative performance assessment based on an average over the SNRs. B, FR calculated for each IVIM parameter for all SNRs

To provide a summary of the quantitative performances of the implemented methods, the metrics were averaged over the SNRs as shown in Figure 7A. Considering the MdAE, the proposed method, on average, outperforms the others for all the IVIM parameters, having an average MdAE of 0.07 ± 0.04 for *D*, 0.14 ± 0.08 for *f* and 0.26 ± 0.1 for D*. As for the average MdB, the proposed method has values of −0.001 ± 0.004 for *D*, −0.006 ± 0.02 for *f* and −0.03 ± 0.005 for D*, outperforming all the others in the case of *D*, *f* and D* estimation.

For the average RCV, the proposed method is the second‐best performing approach for *D* (0.08 ± 0.03), *f* (0.159 ± 0.064) and D* (0.178 ± 0.055).

Finally, focusing on the FR, the proposed method outperformed the others in 9 out of 21 conditions whereas the Bayesian approach prevailed in 11 out of 21. On average, the proposed method has the lowest FRs for the estimation of *f* and D* (13.3% and 24.3% respectively), whereas in the case of *D* the average FR is 1.2%. It is worth noticing that the other AI‐based methods have shown worse performances in all the conditions if compared with the proposed method. Figure 7B shows the FRs in each condition and for each method.

### 3.4 In vivo preclinical images

**FIGURE 8:**
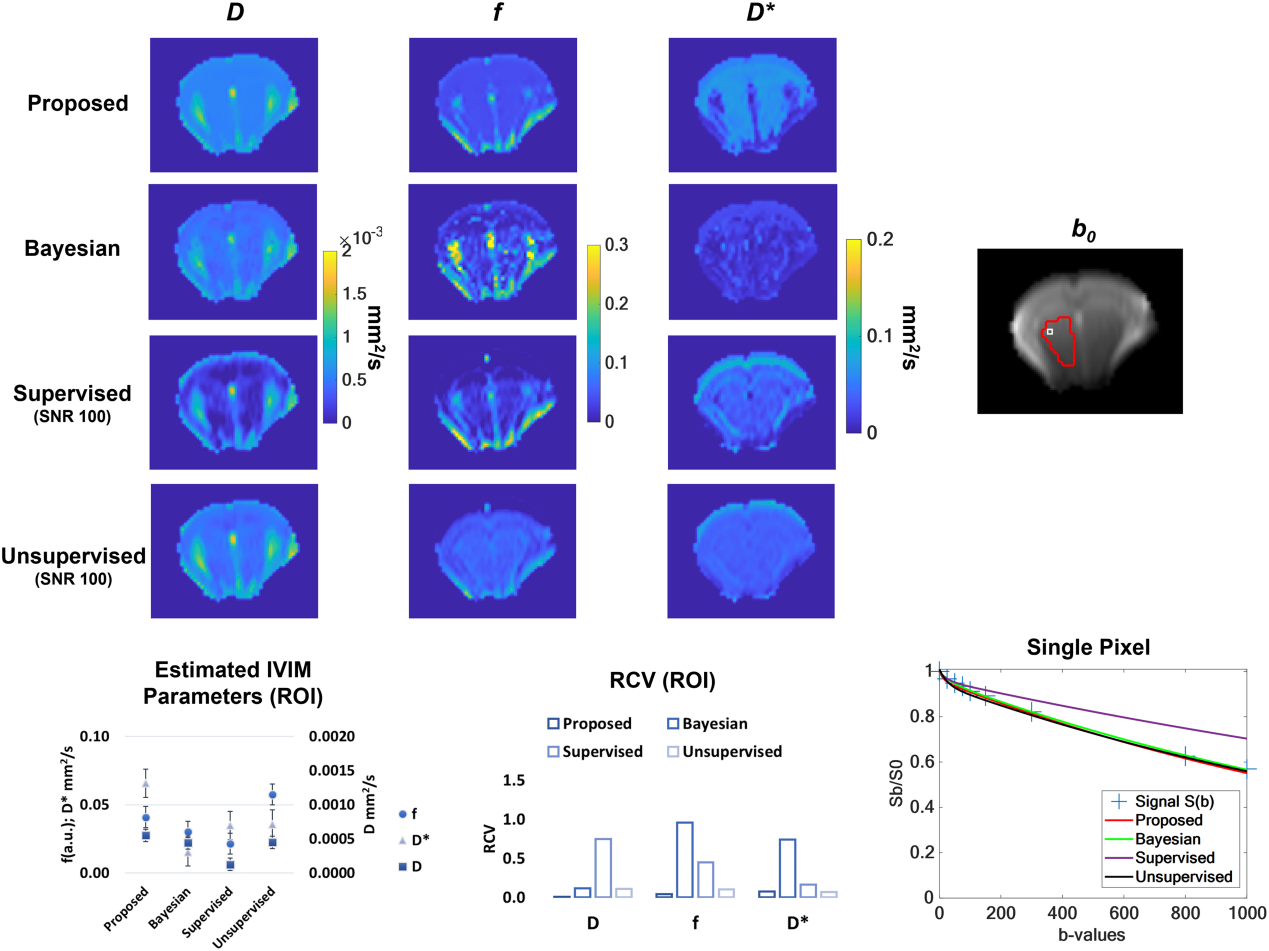
Evaluation of the proposed method, compared with the other implemented approaches, in a real preclinical scenario. The median estimated (±MAD) parameters within a homogeneous ROI along with the median RCV are displayed

The proposed algorithm was tested in a real preclinical scenario and compared with the other methods. Figure 8 shows an example of IVIM parameter maps along with the median values and their RCVs as calculated in a homogeneous ROI. Considering the values of the estimated parameters, the proposed method provided similar results with respect to the Bayesian method in the case of *D* and *f* (*D*_prop_ = 5.5 × 10^−4^ ± 4 × 10^−6^ mm^2^/s, *D*_bayes_ = 4.4 × 10^−4^ ± 3 × 10^−5^ mm^2^/s; *f*_prop_ = 0.04 ± 0.001, *f*_bayes_ = 0.03 ± 0.02), whereas in the case of D* there is an overestimation of the parameter (D*_prop_ = 6.6 × 10^−2^ ± 3 × 10^−3^ mm^2^/s, D*_bayes_ = 1.5 × 10^−2^ ± 1 × 10^−2^ mm^2^/s). Regarding the RCV, the proposed approach outperformed the others for *D* (RCV_prop_ = 0.01), *f* (RCV_prop_ = 0.04) and D* (RCV_prop_ = 0.07) map estimation. In the case of RCV, the Bayesian approach presents the highest values when considering *f* and D* parameters. Considering a single voxel fitting, the proposed method along with the Bayesian and the unsupervised approaches exhibited similar performances, whereas the supervised approach failed to fit the curve, especially at high *b*‐values.

## 4 DISCUSSION

In this work, a novel supervised fully connected DNN aiming at improving IVIM parameter estimation was proposed. The final goal of this work was to provide a method that was ready to use, reliable and easily translatable from research to clinical practice and that overcomes some of the limitations of current methods that are either too inaccurate, too computationally complex and/or too demanding to implement reliably in a clinical or preclinical setting.

The proposed DNN was implemented, tested and compared with other recent Bayesian^25^ and deep learning approaches^21^, ^23^ to evaluate, using simulated DW images and in vivo preclinical images, the overall performance of the network in terms of accuracy and image quality.

The proposed DNN represents an improvement of a previous approach proposed by Bertleff et al.,^21^ which in the original version uses a more complex model containing the kurtosis. Here, the main innovations introduced are (i) the standardization of the target features (i.e., ground‐truth parameters), (ii) the input feature augmentation adding the log‐transformed DW signal as inputs and (iii) the use of a single DNN that was trained with different SNRs. The first two aspects were tested during the tuning phase and provided a substantial improvement in terms of accuracy if compared with the “classical” approach. The parameters we used to test our proposed method showed a consistent accuracy improvement. As expected, the standardization of target provided higher accuracy in *D* maps due to the different order of magnitude of diffusivity compared with D* and *f*.

It is known that feature scaling through standardization (or *Z*‐score normalization) is usually an important preprocessing step for many AI algorithms. An adequate normalization of the input data before network training significantly reduces the estimation errors and the computation times.^27^ The need for scaling target features in a supervised approach is not well defined; however, based on the results described in this paper we suggest that a standardization step can considerably increase the accuracy of the method used. This can be relevant especially when target features have quantitative values that differ by orders of magnitude, as in the case of IVIM parameters.

The approximation capability and result accuracy can increase on adding more input features, even though irrelevant and/or redundant features make learning harder and affect the generalization of learned models.^28^ Due to the complexity of this problem, we used a heuristic approach to decide if adding the natural logarithm of DW signal values as input variables could improve our proposed algorithm. Based on the analysis of MdAE, MdB, RCV and FR, the performance advantages brought by the added log‐transformed features are not negligible.

Eventually, the use of a single trained DNN provides advantages in terms of general usability, especially when compared with the method proposed by Bertleff et al., since our approach removes the reported requirement to replicate training of networks at each different SNR (i.e., practical implementation of Bertleff's approach requires selection of a “compromise” sub‐optimal DNN optimized for a specific SNR level and used to analyze real images at different SNRs). The proposed DNN can be applied to wide SNR ranges from 10 to 100, and depending on the specific application can be retrained to consider even wider ranges, making it ideal for practical implementation when dealing with images from different sources.

The results obtained using simulated data demonstrated that the proposed DNN approach outperforms, in terms of both MdAE and MdB, all the other methods across most SNR conditions. In most cases, the proposed method was less prone to generating outlier IVIM parameter estimates as compared with the other DL methods. As for the RCV, the proposed approach outperforms the unsupervised method in all the conditions and it is better than the Bayesian approach in the case of perfusion‐related parameters (e.g., *f* and D*). In general, based on our performance metrics, the original supervised approach was deemed the worst‐performing method, with our DNN approach exhibiting less failure in IVIM parameter estimation when compared with the other DL approaches.

Even though the proposed DNN works on a voxel‐by‐voxel basis, we opted to employ the Shepp–Logan phantom^29^ image shape for our simulation to achieve a better approximation to realistic in vivo conditions (i.e., heterogeneity of diffusion and perfusion parameter distribution expected in a typical mammalian brain). Additionally, the Shepp–Logan phantom provides an easier and more straightforward visual evaluation of the IVIM reconstruction algorithm performance and quality.

In vivo results demonstrate the feasibility of deploying the described methods for processing of live‐subject IVIM MRI data. The mean estimated parameters obtained in a homogeneous region, for *D* and *f*, are in good agreement with those estimated using the state‐of‐the‐art Bayesian method and the unsupervised approach. As for D*, there is a huge variability among methods and this is due to the high uncertainty in the pseudo‐diffusion estimation, especially when low *D* and *f* values occur, as previously demonstrated with the Bayesian approach.^14^ The outputs from the other DL methods generally over‐ or underestimate the same quantitative parameters. When evaluating RCV, the proposed method was the best performing for all the estimated parameters, thus demonstrating the best precision in a homogeneous region.

The use of a supervised approach adopted in this paper requires a first training step based on simulated data that should be consistent and coherent with the experimental acquired (i.e., real) dataset, from which the IVIM parameters must be obtained. Any alteration in acquisition parameters requires retraining using a different set of simulated data. To overcome this issue, unsupervised approaches, in which the training is directly conducted using real non‐simulated datasets, has been recently proposed and successfully applied.^22^, ^23^ The unsupervised approach, however, requires a much higher number of real experimental acquired data to achieve the objectives, and in addition the network requires retraining if new datasets are included in the study even if the MR protocol (e.g., number of *b*‐values) remains unchanged.

As previously described, estimation of model parameters using supervised ML approaches depends on the training‐set distribution.^30^ In this article a uniform distribution was chosen to sample the IVIM parameters for simulations, and this approach agrees with the other previously published papers.^21^, ^22^, ^23^ Nevertheless, when training data are sampled uniformly, estimates tend to be more accurate but may have lower precision if compared with other sampling strategies.^30^

Considering that the DNN performances might become worse if other modes of noise and artifacts are present in the images (e.g., statistical noise distribution, larger or lower SNRs, motion etc.), it can be necessary to carefully evaluate the available data beforehand and generate numerical simulations that are as realistic as possible to match the characteristics of the real data.

The proposed method was tested on a fixed range of *b*‐values, nine *b*‐values ranging from 0 to 1000 s/mm^2^, which was chosen in agreement with the in vivo DWI protocol. As mentioned, use of a different set of *b*‐values requires a retraining of the DNN in both supervised and unsupervised approaches. It is generally known that sampling with different sets of *b*‐values can affect IVIM parameter estimation performance; however, characterizing the sensitivity and *b*‐value dependence was outside the scope of this paper. To tackle this issue, a supervised DNN approach was recently proposed to simultaneously optimize *b*‐values and estimate IVIM parameters.^31^

Finally, while the supervised and unsupervised training algorithms have similar performances, the supervised learning algorithm offers two main advantages: the highest accuracy and the lowest variability once the DNN is trained. Importantly the above‐described method could be employed to generate parametric results reflective of perfusion status in a non‐invasive manner in organs different from the brain, which often exhibit reduced perfusion conditions when affected by a disease condition (kidney, placenta, etc.).

## 5 CONCLUSIONS

In this work, we provide a flexible supervised DNN ready to be used in a wide variety of scenarios without the need to repeatedly train a new network tailored to each SNR. We demonstrate that the proposed neural network architecture is a valuable alternative tool for the computation of IVIM parameters. Based on the quantitative analysis, the DNN method described in this paper exhibits improved performances if compared with the state‐of‐the‐art Bayesian approach and other DL methods in most of the conditions in simulated scenarios. Considering in vivo data, the feasibility and reliability of the proposed approach are confirmed and the estimated parameters are in agreement with those fitted by the Bayesian and unsupervised approaches, especially for *D* and *f*, but with a lower variability. Based on its performance, the proposed method is promising for in vivo applications even when compared with similar DL approaches.

## Data Availability

The data that support the findings of this study are available from the corresponding author upon reasonable request.
